# Treatment of human oocytes with extracellular vesicles from follicular fluid during rescue *in vitro* maturation enhances maturation rates and modulates oocyte proteome and ultrastructure

**DOI:** 10.1093/hropen/hoag021

**Published:** 2026-03-09

**Authors:** Sofia Makieva, Mara D Saenz-de-Juano, Carmen Almiñana, Stefan Bauersachs, Sandra Bernal-Ulloa, Min Xie, Ana G Velasco, Natalia Cervantes, Maike Sachs, Susanne E Ulbrich, Brigitte Leeners

**Affiliations:** Department of Reproductive Endocrinology, University Hospital Zurich, Zurich, Switzerland; Animal Physiology, Institute of Agricultural Sciences, ETH Zurich, Zurich, Switzerland; Department of Reproductive Endocrinology, University Hospital Zurich, Zurich, Switzerland; Functional Genomics Group, Institute of Veterinary Anatomy, University of Zurich, Zurich, Switzerland; Animal Physiology, Institute of Agricultural Sciences, ETH Zurich, Zurich, Switzerland; Department of Reproductive Endocrinology, University Hospital Zurich, Zurich, Switzerland; Department of Reproductive Endocrinology, University Hospital Zurich, Zurich, Switzerland; Department of Reproductive Endocrinology, University Hospital Zurich, Zurich, Switzerland; Department of Reproductive Endocrinology, University Hospital Zurich, Zurich, Switzerland; Animal Physiology, Institute of Agricultural Sciences, ETH Zurich, Zurich, Switzerland; Department of Reproductive Endocrinology, University Hospital Zurich, Zurich, Switzerland

**Keywords:** follicular fluid, extracellular vesicles, *in vitro* maturation, human oocyte, single-cell proteomic analysis

## Abstract

**STUDY QUESTION:**

Could follicular fluid-derived extracellular vesicles (ffEVs) benefit human oocyte rescue *in vitro* maturation (rIVM)?

**SUMMARY ANSWER:**

Supplementation of rIVM culture with ffEVs isolated from mature follicles enhanced oocyte maturation rates by >20%, inducing changes in oocyte protein profile and organelle distribution.

**WHAT IS KNOWN ALREADY:**

IVM involves the culture of immature germinal vesicle (GV) oocytes under set laboratory conditions to allow for their transition to mature metaphase II (MII) stage, which is confirmed by the extrusion of the first polar body. Efficient IVM could circumvent controlled ovarian stimulation (COS), reduce the cost and broaden the repertoire of infertility treatments. Animal studies suggest that extracellular vesicles (EVs), membranous nanosized vesicles containing different molecular content (e.g. nucleic acids, proteins) and present in the ovarian follicular fluid, could enhance oocyte maturation. The uptake of ffEVs by bovine, equine, and feline oocytes, but not human, has been demonstrated.

**STUDY DESIGN, SIZE, DURATION:**

Women undergoing transvaginal oocyte retrieval after COS (n = 83) were recruited to donate follicular fluid (n = 54 single follicles) and/or immature GV oocytes (n = 95). We aimed to: (i) define differences in the protein cargo of ffEVs derived from human follicles containing mature (MII-ffEVs, n = 10) versus immature (GV-ffEVs, n = 5; metaphase I MI-ffEVs, n = 5) oocytes, (ii) demonstrate the capacity of human GV oocytes to uptake MII-ffEVs and (iii) determine the effect of MII-ffEVs supplementation on oocyte maturation.

**PARTICIPANTS/MATERIALS, SETTING, METHODS:**

ffEVs were isolated by ultracentrifugation. The protein content of ffEVs was analysed by mass spectrometry. The uptake of fluorescently-labelled MII-ffEVs by GV oocytes (n = 15) was assessed by confocal microscopy. GVs were cultured for rIVM in a timelapse incubator with MII-ffEVs (n = 45 GVs) or without (n = 40 GVs), and extrusion of polar body denoted maturation. The impact of MII-ffEVs supplementation on IVM-matured oocytes was assessed through single-cell proteomics and the appearance of intracellular organelles upon transmission electron microscopy (TEM) analysis.

**MAIN RESULTS AND THE ROLE OF CHANCE:**

We identified 1340 proteins in ffEVs, with proteins such as F12, IGKV1-39, FREM2, and C1QC being significantly enriched in MII-ffEVs. GV oocytes internalized MII-ffEVs, and their supplementation for 48 h increased the oocyte maturation rate compared to control by 22.8 ± 9.4% (77.8% vs 55% maturation rate respectively; *P*-value = 0.0372). Proteomic analysis of ffEV-supplemented mature oocytes (n = 5) revealed 56 differentially abundant proteins (DAPs) compared to non-supplemented mature oocytes (n = 5). Among them, 37 DAPs were in higher abundance in ffEVs-supplemented mature oocytes, including Hyaluronan Synthase 1 (HAS1) that is associated with oocyte maturation. Electron microscopy showed differences in oocyte organelle distribution and appearance, particularly that of endoplasmic reticulum (ER) and ER–mitochondria complexes. Functional enrichment analysis of differentially abundant proteins during ffEV–oocyte interaction revealed regulation of endoplasmic reticulum, steroid biosynthesis, and keratin organization pathways.

**LARGE-SCALE DATA:**

Proteomics data are available via ProteomeXchange with identifier PXD073018.

**LIMITATIONS, REASONS FOR CAUTION:**

This study utilized immature oocytes from COS cycles; therefore, the results should be interpreted within the context of rIVM potential. The employed oocytes were vitrified and warmed, and the rIVM was performed for 48 h.

**WIDER IMPLICATIONS OF THE FINDINGS:**

These results provide new insights into the role of ffEVs in enhancing oocyte maturation, offering potential improvements for clinical rIVM protocols, and inspiring the development of global IVM supplements based on ffEVs or associated specific cargo.

**STUDY FUNDING/COMPETING INTEREST(S):**

This work was funded by an EMDO research fellowship and a FAN research grant (Fonds zur Förderung des akademischen Nachwuchses) from the University of Zurich. The authors declare no competing interests.

WHAT DOES THIS MEAN FOR PATIENTS? Infertility rates are rising, with 17% of couples worldwide needing help to get pregnant, often through treatments like IVF. IVF usually involves using hormones to stimulate the ovaries to produce multiple eggs, which can be tough on a woman’s health, both physically and emotionally, and can be very expensive. *In vitro* oocyte maturation (IVM) is a gentler alternative, where eggs are matured outside the body, reducing risks and costs. However, IVM is not as effective as IVF yet, mainly because the current methods are not perfect. Our research is exploring a new approach to improve rescue IVM by adding extracellular vesicles from follicular fluid to the egg culture. This could help the oocytes mature better, leading to higher success rates and giving more options to couples struggling with infertility.

## Introduction

Global fertility rates are falling below replacement thresholds in over half of all countries as of the year 2021; a decline that is anticipated to exert a significant influence on both economic and societal dynamics worldwide (Bhattacharjee *et al.*, 2024). Presently, infertility poses a formidable challenge for one in six couples globally, necessitating recourse to medically assisted reproduction treatments (Harris, 2023). Of these, the most prominent intervention is controlled ovarian stimulation (COS) utilizing exogenous hormones aimed at growing multiple oocytes within small ovarian antral follicles (Macklon *et al.*, 2006). After COS, mature oocytes are retrieved from large follicles to undergo IVF. Despite the rigor of protocols, approximately 15–30% of oocytes remain immature and ineligible for IVF (Mandelbaum *et al.*, 2021). This percentage increases with milder COS regimes, a prerequisite for sensitive groups, such as cancer patients and subfertile women at risk of ovarian hyperstimulation syndrome (Nargund *et al.*, 2022). Even milder stimulation approaches profoundly influence a couple’s experience undergoing infertility treatment (Njagi *et al.*, 2023). For instance, COS necessitates the purchase of expensive hormones and frequent appointments to evaluate follicular growth. Furthermore, the process is not devoid of symptoms; it can induce physical discomfort and even be mentally taxing.

Several advancements aim to reduce the impact of COS. Among these is *in vitro* maturation (IVM), wherein multiple immature oocytes are retrieved from small antral follicles without prior COS and cultured extracorporeal in the laboratory (Gilchrist *et al.*, 2024). Indeed, IVM enhances patients’ satisfaction by streamlining the treatment and reducing risks. This approach benefits not only patients who necessitate mild COS, comprising 15% of the IVF population, but also those with complete resistance to hormones (Vuong *et al.*, 2020; Le *et al.*, 2021). However, despite 80 years of the IVM concept, extensive livestock applications and clinical success, healthcare providers remain wary of adopting a method they perceive as a significant departure from the current convention, namely COS (Rock and Menkin, 1944; Edwards, 1965; Merton *et al.*, 2003; Roesner *et al.*, 2017; Viana, 2020; Guo *et al.*, 2023). Consequently, the clinical integration of IVM has been slow. It has particularly been hindered by the absence of standardisation and efficacy concerns, leading to its classification as non-experimental only in 2021 (De Vos *et al.*, 2021; Krisher, 2022; Makieva *et al.*, 2024). Undermining IVM’s credibility further, oocytes matured *in vitro* are known to possess lower competence compared to their intracorporeally matured counterparts (De Vos *et al.*, 2021). This disparity has also been highlighted by the only two clinical trials comparing the outcomes of IVM to COS, revealing lower cumulative live birth rates for the former (Vuong *et al.*, 2020; Zheng *et al.*, 2022). Nonetheless, IVM has achieved live birth rates exceeding 40% per IVF cycle in selected patients (Ho et al., 2019; Mackens et al., 2020; Mostinckx et al., 2024).

Currently, there are about 10 different IVM variations in clinical usage. Refining these protocols is paramount for maximizing efficacy and addressing concerns (Gilchrist *et al.*, 2024). In conventional or standard IVM (sIVM), immature oocytes with a germinal vesicle (GV) present in the cytoplasm are retrieved from small follicles and maintained surrounded by supportive cumulus cells, forming the cumulus–oocyte complex (COC). Following exposure to hormones for up to 36 h, the COCs are denuded, and oocyte maturity is confirmed by the presence of a polar body, denoting completion of nuclear maturation and oocyte arrest at the metaphase II stage of meiosis I. Conversely, rescue IVM (rIVM) is used to mature denuded GV oocytes retrieved after COS. The addition of hormonal supplements during rIVM is unnecessary due to the demonstrated unresponsiveness to stimulation; therefore, these oocytes are cultured in a standard IVF medium with the hope of maturing spontaneously within 24 h. Indeed, most species’ oocytes, including humans—albeit at a lower frequency—could spontaneously resume meiosis once freed from follicles (Edwards, 1965). Given its seamless integration into conventional protocols and recent supportive findings regarding oocyte competence, rIVM may gain greater priority and acceptance in fertility clinics (Esbert *et al.*, 2024). Successful implementation of rIVM has reportedly increased the pool of available oocytes for treatment while maintaining the integrity of conventional procedures and has led to healthy live births (Shani *et al.*, 2023; Yuan *et al.*, 2024).

Preclinical animal studies suggest a regulatory role for extracellular vesicles from follicular fluid (ffEVs) in governing oocyte maturation, highlighting their potential relevance to IVM (Pioltine *et al.*, 2020a). The ffEVs, nano/micro-scale structures encapsulating nucleic acids and proteins, are released by various cells within the follicle and are considered indicators of oocyte competence and maturity (Santonocito *et al.*, 2014). Studies on diverse animal species have documented the internalisation of ffEVs by oocytes, directly facilitating maturation (de Almeida Monteiro Melo Ferraz et al., 2020; Uzbekova et al., 2020). Our study aimed to investigate for the first time whether supplementation of human rIVM with ffEVs originating from mature MII oocyte-containing follicles could enhance GV oocyte maturation. Using follicular fluid and GV oocytes from women undergoing COS, our objectives included disclosing the ‘mature ffEVs’ protein signature and determining whether the uptake of those ffEVs would increase the maturation rate of GV oocytes in an rIVM system.

## Materials and methods

### Human samples

Follicular fluid samples (n = 54) and immature oocytes (n = 95) were obtained from 83 women undergoing routine oocyte retrieval procedures for infertility treatment at the IVF center of University Hospital Zurich following written informed consent. The women recruited in our study had no previous diagnosed endocrine pathology and were undergoing fertility treatment for oocyte cryopreservation or unexplained infertility. The average age of the women was 36.4 ± 2.7 years. All women received COS with gonadotrophins for up to 14 days to allow the simultaneous growth of multiple ovarian follicles before being triggered for superovulation. Oocyte retrieval took place 36 h after triggering ovulation. Oocytes were denuded from cumulus cells for the purpose of ICSI treatment or oocyte cryopreservation for social purposes. Oocytes that failed to mature despite COS were not used for treatment and, thus, were donated for research. The use of the aforementioned human material has been approved by the local ethics committee (BASEC-Nr. 2018-00797).

### Controlled ovarian stimulation (COS)

Prior stimulation women received a gestagen [10 mg/d] for 10 days up to 28 days, beginning at the second cycle day, in the short or antagonist protocols, and a GnRH-agonist [triptorelin, 0.1 mg/d] on cycle day 21 for the long protocol of COS. For COS, either short or long GnRH-agonist or GnRH-antagonist protocols were used with either hMG or recombinant FSH application. When at least three follicles with a diameter of ≥17 mm were observed during vaginal ultrasound, the final oocyte maturation was induced with either 6500 IE hCG or with the addition of a GnRH-agonist accompanied by about 1600 IE hCG within the GnRH-antagonist protocol. Ultrasound-guided oocyte retrieval was performed around 36 h after administration of the hCG/GnRH-antagonist trigger.

### Oocyte handling

All COCs were cultured in a humidified incubator with conditions of 37°C, 20% O_2_, and 6% CO_2_ in suitable fertilisation media such as Global for Fertilisation (CooperSurgical, Trumbull, CT, USA) or G-IVF (Vitrolife, Gothenburg, Sweden) under oil overlay (OVOIL, Vitrolife). Two hours after retrieval, the oocytes were denuded using hyaluronidase enzyme (80 IU/ml). The denuded immature oocytes were subjected to cryopreservation using the Cryotop^®^ method according to the Kitazato VT601 vitrification protocol (Kitazato, Fuji, Japan) and stored in liquid nitrogen. In this method, an open vitrification carrier, which contains a polypropylene strip accompanied by a protective cover, is used. By aspirating the excess solution that is placed on the filmstrip, only a thin layer covering the cryopreserved cells ultimately remains. Using this minimal volume increases the cooling rate to 2300°C/min and the warming rate to 4210°C/min (Kuwayama, 2007). The oocytes were warmed for the experiments in this study using the VT602 thawing kit (Kitazato). Following warming, the immature oocytes were incubated in fertilisation media under an oil overlay for up to 1 h before being used for experiments.

### Extracellular vesicle isolation from follicular fluid samples

The follicular fluid from single follicles was collected in preheated tubes kept at 37°C and placed in petri dishes for oocyte search. Once the COC was removed, the follicular fluid devoid of blood contamination was collected in tubes, centrifuged at 800*g* for 10 min, and the supernatant was stored at −80°C until further processing. Extracellular vesicles were isolated using differential centrifugation and ultracentrifugation. The follicular fluid samples were thawed on ice and centrifuged at 2000*g* for 30 min at 4 °C to remove cell debris. The supernatant was then transferred into 6.5 ml Ultraclear ultracentrifugation tubes (Beckman Coulter, Nyon, Switzerland) and centrifuged at 110 000*g* at 4°C for 70 min in an Optimax XE90 ultracentrifuge (Beckman Coulter) with a 50.2 Ti rotor (Beckman Coulter). The pelleted ffEVs were washed with sterile filtered PBS (Thermo Fisher Scientific, Reinach, Switzerland) and ultracentrifuged again at 110 000*g* at 4 °C for 70 min.

All procedures before and after ultracentrifugation were performed under the sterile hood to avoid external contamination of ffEVs. Depending on the downstream experiment, final ffEVs were collected in PBS or culture media.

### TEM analyses of ffEVs and oocytes

Extracellular vesicles and oocyte observations were performed by the Scientific Center for Optical and Electron Microscopy (ScopEM, Zurich, Switzerland) of Zurich Eidgenössische Technische Hochschule (ETH) Zürich, Switzerland. For the TEM analysis of ffEVs, three microliters of the vortexed dispersion of EVs were placed on glow-discharged carbon-coated grids (Quantifoil, Großlöbichau, Germany) for 1 min. Negative contrast staining was performed in 2% sodium phosphotungstate at pH 7.2 for 1 s, followed by a second step for 15 s. Excess moisture was drained with filter paper, and the air-dried grids were imaged using a TEM Morgagni 268 (Thermo Fisher Scientific) operated at 100 kV.

Oocyte ultrastructural analysis was done by TEM on oocytes fixed in 2.5% glutaraldehyde (Polysciences Europe GmbH, Hirschberg an der Bergstrasse, Germany) and 2% formaldehyde (Polysciences Europe GmbH) in 0.1 M phosphate buffer (pH 7.4). To enable easy handling of the samples during the following washing and staining steps, the oocytes were embedded in freshly prepared low-gelling temperature agarose (4%; Carl Roth GmbH, Karlsruhe, Germany). After gelling on ice, small cubes containing one oocyte each were cut from the gelled block and washed three times in 0.15 M sodium cacodylate buffer. Then, the samples were postfixed with 2% osmium tetroxide (Polysciences Europe GmbH) supplemented with 2.5% potassium hexacyanoferrate trihydrate and 2 mM calcium chloride. After washing again in cacodylate buffer, the samples were stained with 2% aqueous osmium tetroxide, 1% aqueous uranyl acetate (Polysciences) and Walton’s lead aspartate (Deerinck *et al.*, 2010). Between the staining steps, the samples were washed in double-distilled water. Then, the samples were dehydrated in increasing concentrations of ethanol and stepwise infiltrated with Epon (2 × 30% Epon in ethanol, 2 × 70% Epon, and 3 × 100% Epon; Epoxy Embedding Kit, Sigma-Aldrich, St. Gallen, Switzerland). Until 70% Epon, all steps were performed in a BioWave Pro+ Tissue Processor (Ted Pella Inc., Redding, CA, USA). The samples were left in 100% Epon for 1 h, 2 h, and 3 h at RT on a shaker, then transferred into fresh Epon resin and polymerized at 60°C for 3 days. Thin sections of 50 nm were obtained with a diamond knife (Diatome Ltd., Nidau, Switzerland) on a Leica UC7 ultramicrotome (Leica Microsystems, Heerbrugg, Switzerland), placed on formvar and carbon-coated TEM grids (Quantifoil), and stained with 2% uranyl acetate and Reynold’s lead citrate. The stained sections were then visualized using a Talos L120C TEM (Thermo Fisher Scientific) equipped with a Ceta-S camera at 80 kV. Image mosaics were recorded to cover the entire cross-section of each oocyte using the MAPS software (Thermo Fisher Scientific).

### Tunable resistive pulse sensing and Western blotting

Particle concentration and size distribution of isolated ffEVs were performed using the qNano Gold system (Izon Science, Christchurch, New Zealand) and an NP400 Nanopore. The measurement was performed with undiluted samples and the CPC400 beads as the calibration standard. The number of particles analysed per sample was >1000. Data were processed with Izon Control Suite software version 3.3 (Izon Science). The protein concentration of ffEVs and western blotting was performed as previously described (Saenz-de-Juano *et al.*, 2022). The protein concentration of ffEVs was measured using the Pierce™ Bicinchoninic Acid (BCA) Protein Assay Kit (Thermo Fisher Scientific) and the NanoDrop 2000 (Thermo Fisher Scientific). Samples of ffEVs diluted in RIPA buffer were mixed with 4× Laemmli Buffer (Bio-Rad, Basel, Switzerland) and incubated for 5 min at 95°C. If reducing conditions were necessary, 10% β-mercaptoethanol (Sigma-Aldrich) was added to the mix. Samples were loaded into a 4–20% Mini-PROTEAN TGX Stain-Free Precast Gel (Bio-Rad), and electrophoresis was performed at 200 V for 30 min. Proteins were transferred onto a 0.2 µm PVDF Trans-Blot Turbo Transfer Pack (Bio-Rad) using the Trans-Blot Turbo Transfer System (Bio-Rad) with transfer conditions of 1.3 A, 25 V, for 7 min. Immediately, the membrane was blocked with TBST containing 5% skimmed milk powder (Sigma-Aldrich) at room temperature for 1 h. The membrane was then incubated overnight with rabbit anti-TSG-101 (1:500, PA531260, Thermo Fisher Scientific), mouse anti-CD63 (1:250, ab59479, Abcam, Cambridge, UK), and rabbit anti-MFGE8 (1:250, HPA002807, Sigma-Aldrich). After overnight incubation, the membrane was washed and incubated with the secondary antibodies: goat anti-rabbit IgG-HRP (1:10 000, sc-2004, Santa Cruz Biotechnology, Dallas, TX, USA) and goat anti-mouse IgM-HRP (1:10 000, sc-2005, Santa Cruz Biotechnology). Precision Protein StrepTactin-HRP (Bio-Rad) was also added to visualize the ladder. Finally, Clarity™ ECL Substrate (Bio-Rad) was applied to the membrane, and bands were visualized with the ChemiDoc™ MP Imaging System (Bio-Rad).

### RNA extraction and miRNA qPCR

RNA cargo from ffEVs was isolated with the miRNeasy MicroKit (Qiagen, Hombrechtikon, Switzerland). The concentration of the RNA was determined using the Quantus™ Fluorometer and the QuantiFluor^®^ RNA System kit (Promega, Dübendorf, Switzerland). The Agilent Pico Kit and the Agilent 2100 BioAnalyzer (Agilent Technologies, Basel, Switzerland) were utilized to assess the length of RNA fragments. We used the TaqMan™ Advanced miRNA Assays (Thermo Fisher Scientific) to evaluate the specific miRNA. A qualitative detection for the four miRNAs in four ffEVs samples from individual follicles was performed. Data were expressed as mean values of the quantification cycle (Cq). We used the TaqMan Advanced miRNA cDNA Synthesis Kit (A28007, Thermo Fisher Scientific) to generate cDNA from total RNA (2 µl of total RNA input), following the manufacturer’s instructions. The abundance of four miRNAs [hsa-let-7a-5p (478575_mir assay, Thermo Fisher Scientific), hsa-let-7b-5p (478576_mir, Thermo Fisher Scientific), hsa-miR-148a-3p (477814_mir, Thermo Fisher Scientific), and hsa-miR-223-3p (477983_mir assay, Thermo Fisher Scientific)] was measured using TaqMan Fast Advanced Master Mix (#4444556, Thermo Fisher Scientific). The real-time PCR reactions were performed in 384-well plates in a final volume of 10 μl. The PCR cycle parameters included 1 cycle of enzyme activation and cDNA denaturation at 95°C for 30 s, followed by 40 cycles of denaturation at 95°C for 5 s and annealing/extension at 60°C for 30 s.

### Analysis of human ffEVs protein cargo by mass spectrometry

The proteome of ffEVs was analysed using liquid chromatography coupled to high-resolution tandem mass spectrometry (LC–MS/MS) at the Functional Genomic Centre Zurich (FGCZ, Zurich, Switzerland). The ffEVs samples were boiled for 10 min at 95°C followed by mechanical lysis using a tissue homogenizer (2 × 2 min cycles at 30 Hz and high-intensity focused ultrasound (HIFU)). Proteins were reduced and alkylated by adding Tris(2-carboxyethyl)phosphine and 2-chloroacetamide to a final concentration of 2 mM and 15 mM, respectively. Then, the samples were incubated for 30 min at 30°C (700 rpm, and light-protected). After dilution with pure ethanol to reach a final concentration of 60% EtOH (v/v), the KingFisher Flex System (Thermo Fisher Scientific) was used to bind the proteins to the carboxylated magnetic beads (hydrophobic and hydrophilic) and wash them. The enzymatic digestion was performed by adding trypsin in 50 mM triethylammonium bicarbonate buffer (TEAB) and incubating the samples overnight at 37 °C. The remaining peptides were extracted from beads with water, and the two elutions were combined and dried down. The digested samples were dissolved in aqueous 3% acetonitrile with 0.1% formic acid, and the peptide concentration was estimated with the Lunatic UV/Vis absorbance spectrometer (Unchained Lab, Zug, Switzerland). Peptides were separated on an M-class UPLC and analysed on an Iontrap mass spectrometer (Thermo Fisher Scientific).

The acquired data were processed using the Fragpipe v17 (Kong *et al.*, 2017). The spectra were searched against the *Homo sapiens* (SwissProt) protein background database, using Acetyl (Protein N-term) and Oxidation (M) as variable modifications and Carbamidomethyl (C) as fixed modifications. The protein identification results were imported for further analysis in the software Scaffold v5.2.0 (Proteome Software Inc., Portland, OR, USA).

### Single oocyte proteomics analysis

The lysis buffer, consisting of 20 μl of 50 mM TEAB and 0.1% DDM (n-dodecyl-β-d-maltoside), was directly added to the oocytes, which were stored in 0.2 ml tubes. Samples were then snap-frozen in liquid nitrogen and heated at 95°C for 10 min. This freeze–heat cycle was repeated three times. Proteins were reduced and alkylated by adding Tris(2-carboxyethyl) phosphine and chloroacetamide to a final concentration of 5 mM and 15 mM, respectively. The samples were incubated for 30 min at 30°C (700 rpm and light-protected). Then, digestion was done in a buffered trypsin solution at pH 8 (10 mM Tris/2 mM CaCl_2_). Samples were enzymatically digested using 2 µl of 4 µg/µl trypsin overnight plus an additional incubation for 3 h the next day with 2 µl of 4 µg/µl Trypsin. Digestion was stopped by acidifying the samples. Peptide concentration was estimated using the Lunatic UV/Vis absorbance spectrometer (Unchained Lab). Consequently, samples were loaded on Evotip (Evosep, Odense, Denmark), following the manufacturer’s instructions. Samples were analysed on an Evosep One LC coupled to a TIMS TOF Pro mass spectrometer (Bruker, Fällanden, Switzerland), acquiring the data in diaPASEF scans.

Independent acquisition spectra were processed with DIA-NN (Demichev *et al.*, 2020) using a library-free approach with the protein database *Homo sapiens* (SwissProt) with one sequence per gene. The modifications set for variable and fixed modifications were Oxidation (M) and Carbamidomethyl (C), respectively. The protein identification and quantification were performed using FragPipe (Demichev *et al.*, 2022). To filter and normalize the data, a set of functions implemented in the R package [prolfqua] was used (Wolski *et al.*, 2023) using R version 4.1.2 (R Foundation for Statistical Computing, Vienna, Austria), accessed in April 2023 in Zurich, Switzerland. The resulting protein matrix was filtered to consider proteins with a minimum of 2 peptides/protein.

### Data mining and bioinformatics analysis of ffEVs protein cargo and oocyte proteomic profiles

Total identified proteins were filtered to select proteins present in at least 4 out of 5 samples in at least one group for oocyte data. For ffEVs data, the identified proteins were filtered to select proteins present in at least 4 out of 5 samples for GV-ffEVs and GVBD-ffEVs, and 9 out of 11 for MII-ffEVs data. Missing value imputation and statistical analysis were also performed with the DEP tool, a BioConductor R-tool for proteomics analysis (Zhang *et al.*, 2018). The qri imputation method was used for oocytes, and the *man* imputation method was used for ffEVs. Subsequently, differential enrichment analysis was performed by applying empirical Bayes statistics on protein-wise linear models using limma (Smyth, 2004; Ritchie *et al.*, 2015).

The mass spectrometry proteomics data have been deposited to the ProteomeXchange Consortium via the PRIDE partner repository with the dataset identifier PXD073018.

BioDBnet tool v2.1 (Mudunuri *et al.*, 2009; https://biodbnet-abcc.ncifcrf.gov/; accessed in April 2023 in Zurich, Switzerland) was used to convert Uniprot protein identifiers to gene symbols and NCBI Entrez Gene IDs. Then, human gene identifiers or symbols were used for subsequent functional annotation. The self-organizing tree algorithm software (SOTA, Multi Experiment Viewer software v.4.8.1, Dana-Farber Cancer Institute, Boston, MA, USA, accessed in January 2024 in Zurich, Switzerland) (Howe *et al.*, 2011) was used to identify clusters of proteins/genes with similar expression profiles across experimental groups.

We used g:Profiler software (https://biit.cs.ut.ee/gprofiler/gost; accessed in January 2024 in Zurich, Switzerland) with default parameters to obtain information about overrepresented biological functions and pathways in DAPs identified in ffEVs or single oocyte datasets. The protein–protein interactions between MII-ffEVs and treated-oocyte DAPs were obtained using STRING v12 (Morris *et al.*, 2018).

### Uptake of labelled ffEVs by oocytes

The ffEVs used for the uptake experiment were isolated from 50 ml of pooled follicular fluid from 10 patients. The ffEV sample was labelled using green Vybrant™ DiO solution dye (Thermo Fisher Scientific). A total of 19 GV oocytes from 4 different patients were thawed and distributed randomly in 1 of the 3 conditions as follows: (i) 10 oocytes we co-incubated with G1 medium with labelled ffEVs, (ii) 5 oocytes were co-incubated with G1 medium with labelled PBS (control dye), and (iii) 4 oocytes were co-incubated with just G1 medium (control). GV oocytes were incubated for 24 h, fixed, and individually evaluated under a confocal microscope Zeiss LSM 780 (Carl Zeiss AG, Feldbach, Switzerland). To perform the ffEV uptake experiments, the ffEV pellet obtained after ultracentrifugation was resuspended in 250 µl of PBS. The resulting ffEV sample had a protein concentration of approximately 950 µg/ml. From this, 200 µl of ffEVs were labeled using green Vybrant™ DiO solution dye (Thermo Fisher Scientific). Briefly, 1 µl of DiO dye was mixed with 200 µl of the ffEV pellet diluted in PBS or 200 µl of PBS (used as a dye control). Both the dye-ffEV and dye-PBS solutions were vortexed and incubated for 20 min at 37°C in the dark. Subsequently, both solutions were centrifuged at 2000 *g* for 5 min at 4°C to remove any potential dye aggregates. The results labelled ffEVs and labelled PBS solutions were resuspended in 50 µl of sterile G1-IVF medium and incubated with oocytes. GV oocytes were incubated for 24 h at 37°C, 20% O_2_, and 6% CO_2_. The next day, oocytes were washed four times in PBS supplemented with 1% polyvinyl alcohol (PVA) and fixed in 4% paraformaldehyde (PFA) for 20 min at 37°C. Afterward, the oocytes were washed three times for 10 min each in PBS with 1% PVA and mounted on a microscope slide using VECTASHIELD PLUS antifade mounting medium with DAPI (H2000-10, Vector Labs, Newark, CA, USA). Confocal images were acquired using a Zeiss LSM 780 confocal laser microscope (Carl Zeiss AG, Feldbach, Switzerland) with the objectives 40× 0.75 NA EC Plan-Neofluar Ph2 M27 and 63× 1.4 NA Oil Plan-Apochromat DIC M27. The acquired images were processed using Zen 2012 Blue Edition software (Carl Zeiss AG).

### In vitro *maturation experiments*

In total, three independent IVM experiments were performed. In each experiment, ffEVs for the *in vitro* treatment were obtained from a pool of follicular fluid (60–80 ml) from 12 to 17 different patients. In each experiment, oocytes at the GV stage were thawed and divided into the ffEV treatment group and control group. The first experiment used 31 oocytes from 17 patients (15 control, 16 ffEVs treatment), the second experiment used 31 oocytes from 22 patients (15 control, 16 ffEVs treatment), and the third experiment used 23 oocytes from 17 patients (10 control, 13 ffEVs treatment). The IVM culture was performed using a timelapse GERI Incubator (Genea Biomedx, Sydney, Australia) to record the time of the polar body extrusion. GV oocytes were incubated in individual microwells of a single GERI dish (Genea Biomedx) sharing 100 µl of G1-IVF medium supplemented with resuspended ffEVs pellet (ffEV treatment group) or in another GERI dish sharing 100 µl of G1-IVF medium (control group), for 48 h, at 37°C and 6% CO_2_. After 48 h, the maturation stage of the oocytes was evaluated individually under the microscope using morphological criteria. Additionally, the exact time of the polar body extrusion was noted using the GERI software (Genea Biomedx). Data on maturation rates and polar body extrusion from three independent experiments were analysed using Fisher’s exact test in GraphPad Prism 9 v.9.2.0 (San Diego, CA, USA). The rIVM-matured oocytes were either individually snap-frozen in liquid nitrogen for proteomic analysis or fixed for TEM evaluation.

## Results

### Successful isolation of ffEVs from pools and individual follicles

**Figure 1.**
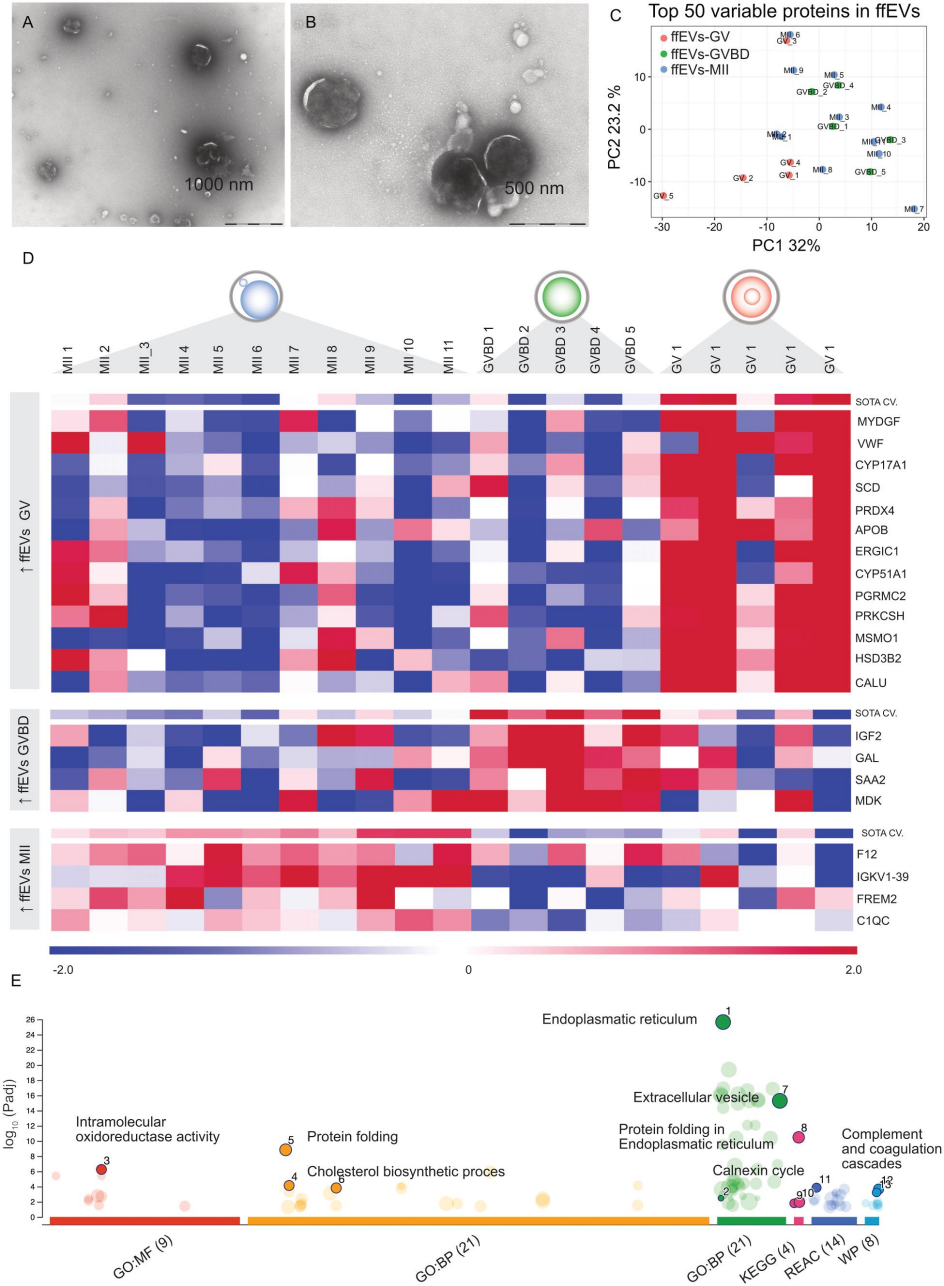
**Follicular fluid extracellular vesicles' protein cargo changed during oocyte maturation.** (**A and B**) TEM analysis confirmed that follicular fluid extracellular vesicles (ffEVs) were successfully isolated and exhibited size heterogeneity. (**C**) Principal component analysis (PCA) based on the top 50 proteins with the greatest changes between all experimental groups (ffEVs-MII, ffEVs-GVBD, and ffEVs-GV). (**D**) Heatmap showing differential abundant proteins (DAPs) in ffEVs (FDR < 0.1). Self-organizing tree algorithm (SOTA) analysis identified 3 clusters of proteins with similar expression profiles across different maturation stages. (**E**) GOSt multiquery plot of Manhattan showing gene ontology (GO) overrepresentation analysis results of 76 unique proteins in MII-ffEVs. BP, biological process; CC, cellular component; GV, germinal vesicle; GVBD, germinal vesicle breakdown, KEGG, Kyoto Encyclopedia of Genes and Genomes; MF, molecular function; MI, metaphase I; MII, metaphase II; REAC, reactome; WP, Wiki pathways.

Transmission electron microscopy (TEM) analysis confirmed the presence of heterogenic ffEVs populations in individual follicular fluid samples (Fig. 1A and B). The size distribution evaluation with tunable resistive pulse sensing (TRPS) unveiled particle dimensions ranging from 50 to 700 nm (Supplementary Fig. S1A). We successfully extracted measurable quantities of RNA from ffEVs derived from 1.5 to 2.0 ml of 4 patient-individual follicular fluid samples and obtained an average of 3.64 ± 2.12 ng RNA. The Bioanalyzer profiles showed that the majority of the RNA belonged to the small RNA population, with a minimal contribution from larger RNAs (Supplementary Fig. S1B). Furthermore, within the isolated ffEVs, we identified the presence of previously reported follicular fluid EV-miRNAs (Martinez *et al.*, 2018) (Supplementary Fig. S1C). Immunoblotting results confirmed three known EV markers, including transmembrane proteins CD63 (Supplementary Fig. S1D), cytosolic protein tumour susceptibility gene 101 (TSG101, Supplementary Fig. S1E), and milk fat globule-EGF factor 8 protein (MFGE8, Supplementary Fig. S1F) in isolated ffEVs. Proteomic analysis by mass spectrometry of ffEVs derived from a pool of follicular fluid samples from different patients revealed that ffEVs also contained further exosomal protein markers such as CD81, CD9, FLOT1, FLOT2, and other proteins characteristic of EV biogenesis, such as annexins, heat shock proteins, and Rab GTPases proteins (Supplementary Table S1).

### Differential protein cargo of human ffEVs derived from follicles containing mature or immature oocytes

We proceeded to analyse the proteomic cargo of ffEVs to identify proteins associated with maturation. Hence, we isolated ffEVs from single follicles containing a mature MII oocyte (n = 11 patients; 1 ffEVs sample per patient), intermediate maturity GVBD (germinal vesicle breakdown) oocyte (n = 5 patients; 1 ffEVs sample per patient), and immature GV oocyte (n = 5 patients; 1 ffEVs sample per patient) to compare their proteomic cargo.

A total of 1340 proteins were identified across all ffEVs samples, regardless of their origin (Supplementary Table S2). Only proteins present in at least 9 out of 11 for the MII group and proteins present in 4 out of 5 GV or GVBD samples were selected for further statistical analysis, resulting in 789 filtered proteins (Supplementary Table S3).

A principal component analysis (PCA) based on the top 50 proteins with the greatest changes across the dataset showed a dynamic protein ffEV pattern, with GV-ffEVs samples separated from the rest of the samples based on principal component 1 (Fig. 1C). To reveal differential abundant proteins (DAPs), three comparisons were statistically performed: MII versus GV, GVBD versus MII, and GVBD versus GV, resulting in 14, 7, and 1 DAPs, respectively (False discovery range; FDR < 0.1; Supplementary Table S4). The DAPs resulting from the three comparisons were further analysed for their expression profiles across samples using a SOTA clustering analysis. This analysis provided three clear clusters (Fig. 1D): cluster 1, with 13 proteins more abundant in GV-ffEVs; cluster 2, with 4 proteins more abundant in GVBD-ffEVs; and cluster 3, with 4 proteins more abundant in MII-ffEVs. The proteins exclusively associated with the MII-ffEVs cargo were the Coagulation Factor XII (F12), Complement C1q subcomponent subunit C (C1QC), FRAS1-related extracellular matrix protein 2 (FREM2), and Immunoglobulin Kappa Variable 1-39 (IGKV1-39).

When a cut-off of FDR < 0.2 was used, 61, 15, and 14 DAPs were identified in the comparisons MII versus GV, GVBD versus MII, and GVBD versus GV, respectively (Supplementary Table S5). A short-listing of the DAPs specific to the MII ffEVs identified 76 unique proteins. These proteins were used for the overexpression analysis using g:Profiler. A functional enrichment analysis highlighted that these DAPs were mainly related to pathways involving endoplasmic reticulum, oxidoreductase activity, complement cascade, extracellular vesicles, and steroid biosynthesis (Fig. 1E; Supplementary Table S6).

### Immature oocytes uptake MII-ffEVs

**Figure 2.**
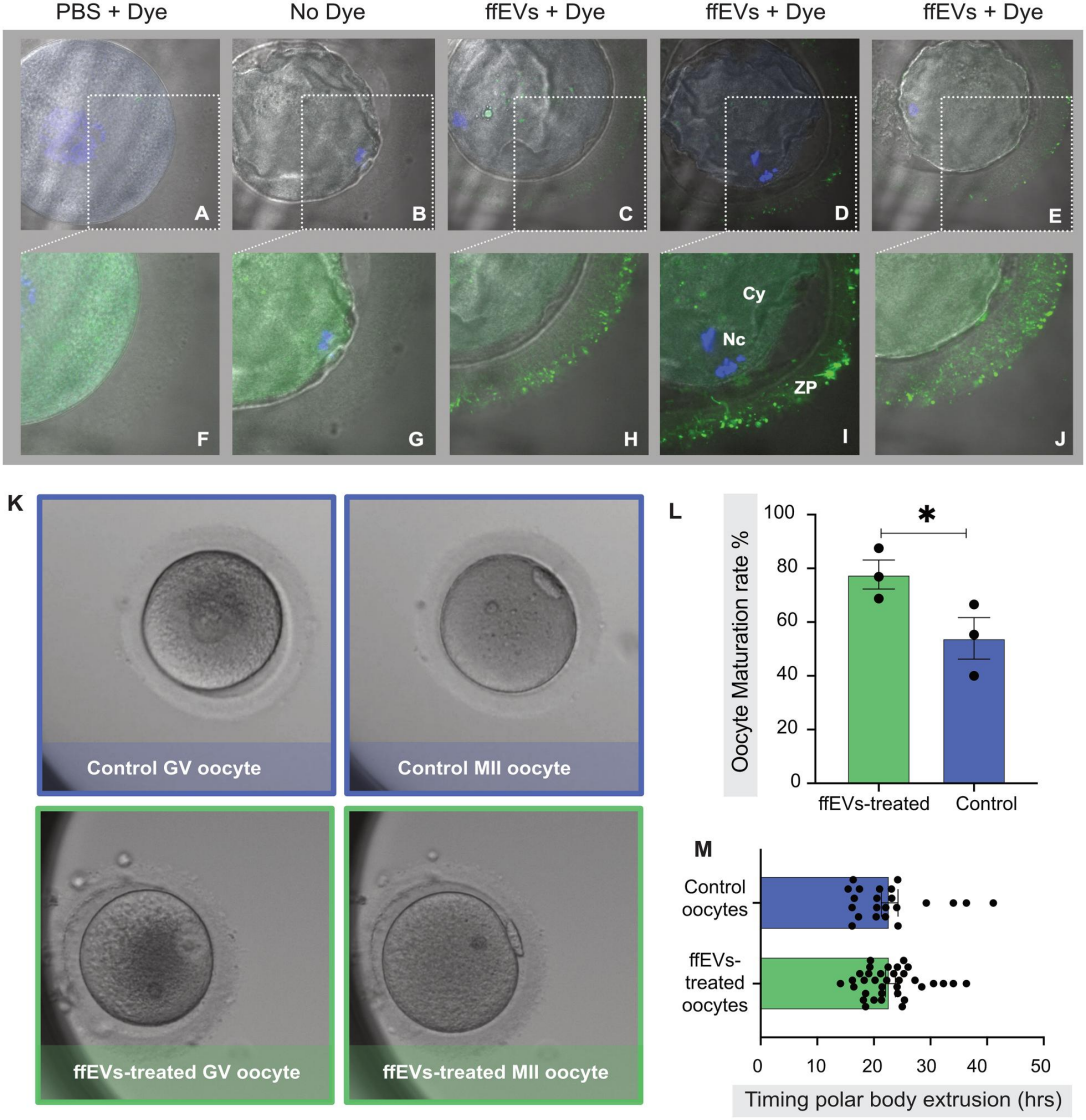
**The uptake of metaphase II-follicular fluid extracellular vesicles (MII-ffEVs) by immature germinal vesicle (GV) oocytes enhanced maturation rates.** GV oocytes were cultured for 24 h under three different treatment conditions: PBS + Dye (PBS labelled with Dye to act as control) (**A**), no treatment (No Dye control; **B**) and with labelled MII-ffEVs (ffEVs+Dye, **C, D, and E**). The insets in the top panel of oocyte images (A, B, C, D, and E) are magnified in the bottom panel (**F, G, H, I, and J**). The bottom panel was acquired during confocal imaging using a higher exposure on the green channel to allow for clear visualisation of the transzonal projections, which are structures in the ZP that function to maintain bidirectional communication between oocytes and cumulus cells. Cy, cytoplasm; Nc, nuclei; ZP, zona pellucida. (**K**) GV oocytes were cultured in a time-lapse Geri incubator for 48 h with or without ffEVs originating from mature follicles. At the end of the culture, GV oocytes either arrested at the immature state or progressed to an intermediate state of maturity (GVBD) or completely matured (MII). Mean ± SD of oocyte maturation rates (**L**) and polar body extrusion time (**M**) in ffEVs-treated and control groups after three independent experiments. Data were analysed using Fisher’s exact test. An asterisk (*) denotes significant differences (*P* < 0.05) in the data.

Since we observed that certain proteins were enriched in ffEVs coming from mature follicles, we sought to examine whether immature oocytes could take up the ‘maturity’ signal by internalizing ffEVs. For studying the outcome of this internalisation, we co-incubated GV immature oocytes with fluorescently labelled ffEVs (coming from mature follicles) for 24 h and performed confocal microscopy to identify any fluorescent signal inside the oocytes. Indeed, we found that oocytes had uptaken the ffEVs. Many localized in the zona pellucida and perivitelin space (Fig. 2A–J), the glycoprotein layer that functions as a mediator between the oocyte and the cumulus cells, which nourish the oocyte during *in vivo* oocyte maturation. It is possible that oocytes recruit the same mechanism to uptake ffEVs as the one used to internalize nutrients from cumulus cells.

### Incubation of GV oocytes with MII-ffEVs during rIVM enhances maturation rates

Following the confirmation that immature GV oocytes internalized ffEVs, we quantified the impact of this internalisation by examining the oocyte maturation rate. We cultured GV oocytes in a time-lapse incubator for 48 h with or without ffEVs. Using time-lapse imaging, we registered the timing of the polar body extrusion and the number of mature oocytes (MII) at the end of the culture in each group (Fig. 2K). The maturation of each oocyte was confirmed under bright light microscopy before snap-freezing for single-oocyte proteomics or fixation for TEM. In three independent experiments, we found that the supplementation of culture media with ffEVs significantly increased the oocyte maturation rate by an average of 22.8 ± 9.4% (*P* = 0.037; Fig. 2L). Specifically, treated ffEVs oocytes displayed 77.7 ± 5.4% (average ± SEM) maturation compared to 54.0 ± 7.7% of controls. The timing of polar body extrusion was comparable between the two groups (22.9 ± 0.8 and 22.8 ± 1.4 h for ffEVs-treated oocytes and controls, respectively; Fig. 2M).

### Treatment with MII-ffEVs changes the proteome of oocytes

**Figure 3.**
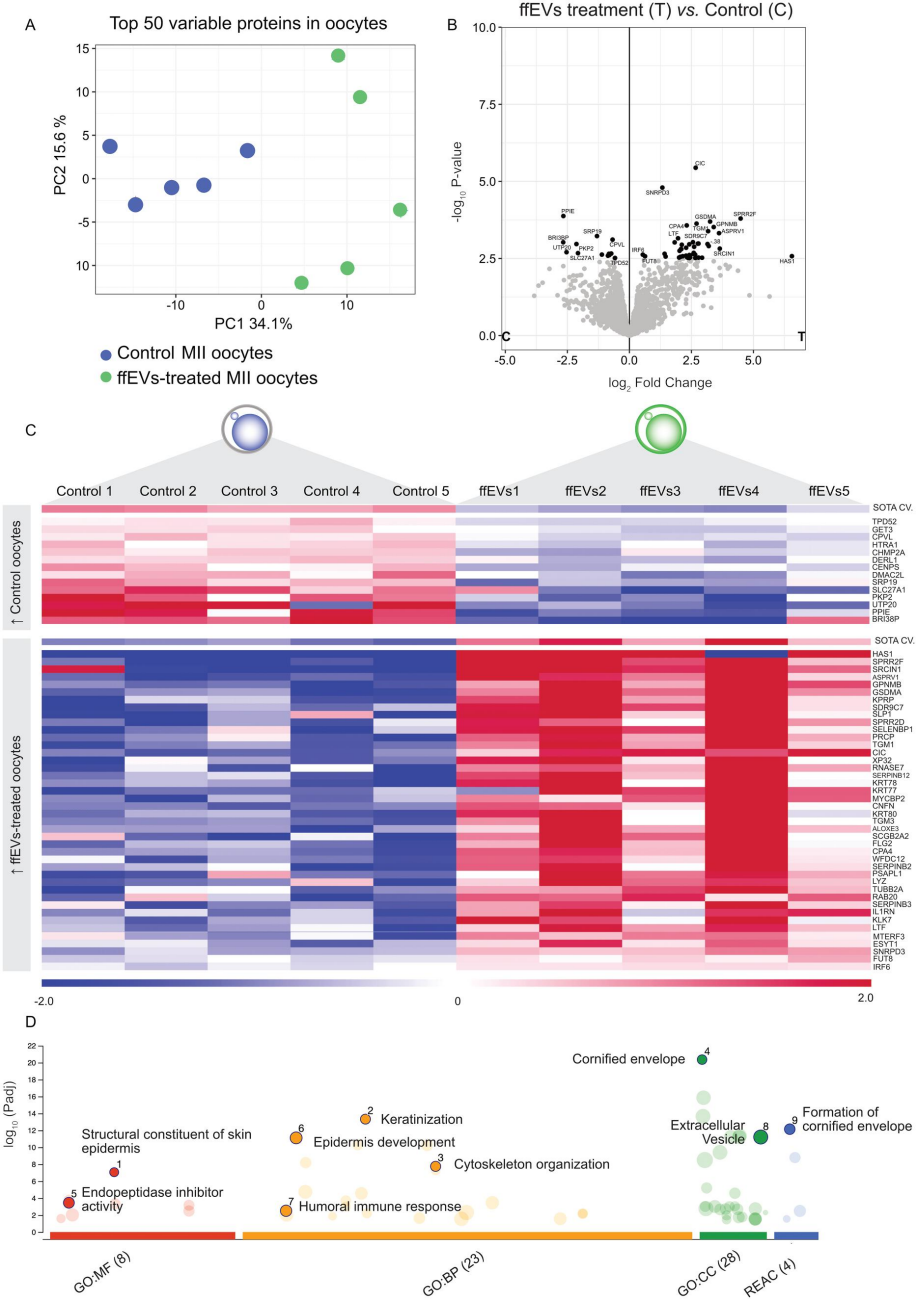
**Follicular fluid extracellular vesicles (ffEVs) treatment modulated the oocyte proteome after maturation.** (**A**) Principal component analysis (PCA) based on the top 50 proteins with the greatest change between all experimental groups (ffEVs-treated oocytes n = 5, control oocytes n = 5). (**B**) The volcano plot illustrates significantly differentially abundant proteins (DAPs) (FDR < 0.1). The −log_10_  *P*-value is plotted against the log_2_ fold change: ffEVs-treated/control. (**C**) Heatmap showing DAPs in ffEVs (FDR < 0.1). Self-organizing tree algorithm (SOTA) analysis identified two clusters of proteins with similar expression profiles. (**D**) GOSt multi-query plot of Manhattan showing gene ontology (GO) overrepresentation analysis results of 76 unique proteins in MII-ffEVs. BP, biological process; CC, cellular component; MF, molecular function; REAC, reactome.

Mature oocytes cultured with or without ffEVs were subjected to single oocyte proteomics. A total of 4587 proteins were identified across all samples (Supplementary Table S7). For the downstream analysis, these proteins were filtered, and only proteins present in at least 4 out of 5 control or treated oocyte samples were selected, resulting in 3884 filtered proteins (Supplementary Table S8). A PCA based on the top 50 proteins with the greatest changes across the dataset showed that samples were distributed in 2 groups (Fig. 3A). Statistical analysis revealed 56 DAPs (FDR < 0.1, Fig. 3B; Supplementary Table S9). The highest difference was found in Hyaluronan Synthase 1 (HAS1) with an increased fold-change of 6.6 in ffEVs-treated oocytes. These DAPs were further analysed for their expression profiles across samples using SOTA clustering analysis, which provided two clear clusters (Fig. 3C). Cluster 1, with 15 proteins, showed increased abundance in control mature oocytes, while cluster 2, with 44 proteins, represented increased abundance in ffEVs-treated mature oocytes.

With a cut-off of FDR < 0.2, 93 DAPs emerged from the latter comparison (Supplementary Table S10). These DAPs were used for the GO overexpression analysis using g:Profiler functional enrichment and were mainly related to pathways involving keratinization, cytoskeleton organization, endopeptidase inhibitor activity, humoral immune response, and extracellular vesicle (Fig. 3D; Supplementary Table S11).

**Figure 4.**
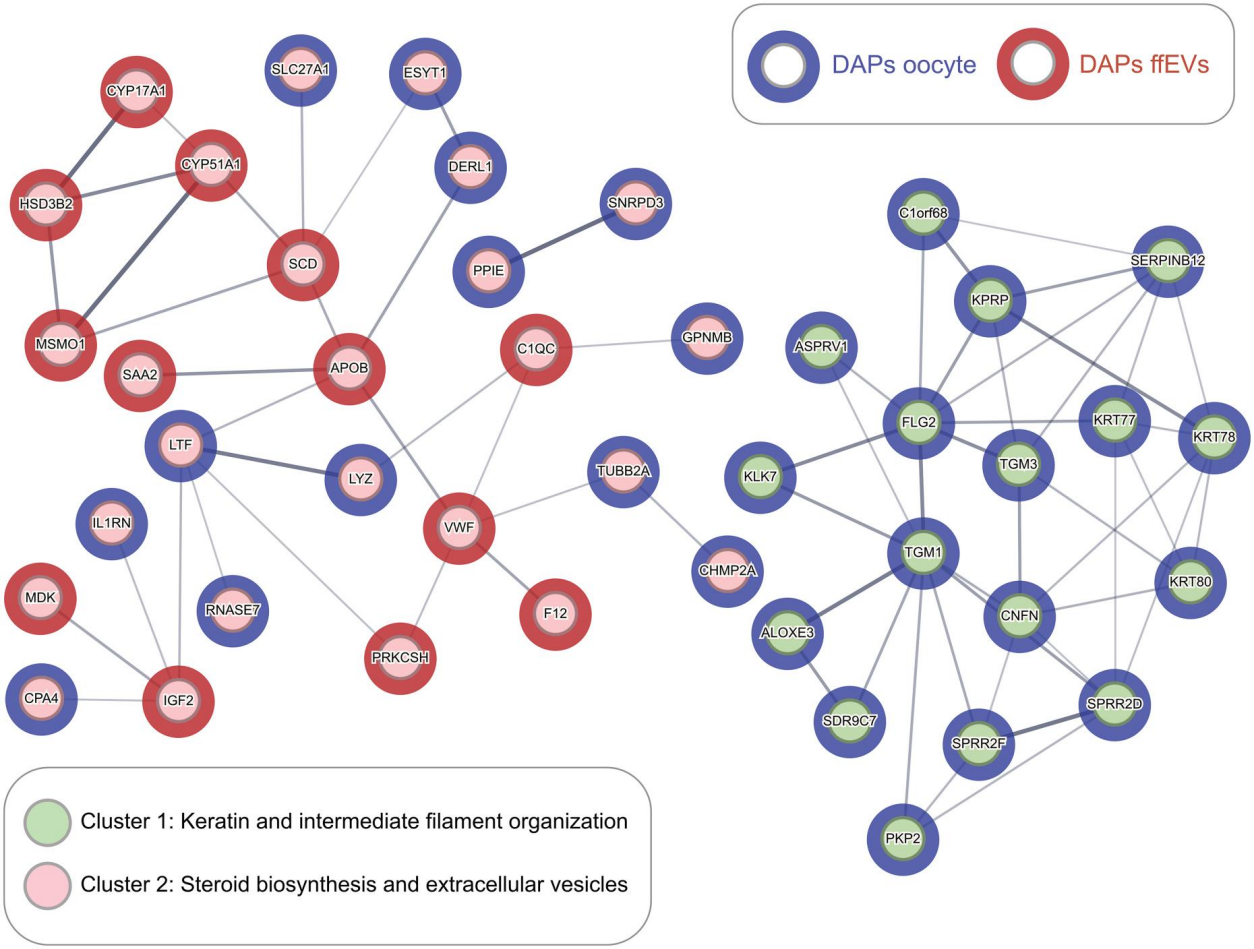
**Oocyte and follicular fluid extracellular vesicles (ffEVs) differentially abundant proteins (DAPs) communication network in rIVM.** DAPs identified as specific to mature or immature ffEVs and DAPs identified in oocytes following rIVM with ffEVs were subjected to a pooled analysis to reveal possible interactions between proteins in the oocytes and proteins in ffEVs. Proteins are shown as nodes connected by lines that signify interacted, and line thickness indicates the strength of the data support. Blue nodes represent oocyte DAPs, and red nodes represent MII-ffEV DAPs. Two clusters can be observed (cluster 1—green, and cluster 2—pink). The green cluster is only composed of oocyte-originating proteins relevant to keratin and intermediate filament organization, while the pink cluster contains proteins from both the oocyte and the ffEVs relevant to steroid synthesis, lipid metabolism, and extracellular vesicles. MII, metaphase II.

To explore how the DAPs identified in ffEVs and among mature oocytes might be connected, we created a protein network using STRING and visualized possible interactions between proteins originating from ffEVs or oocytes (Fig. 4). We observed two noteworthy clusters, one exclusively composed of proteins originating from the oocyte DAPs (cluster 1, green) and another composed of proteins originating from both ffEVs and oocytes (cluster 2, pink). Functional enrichment analysis revealed that cluster 1 was related to keratin and intermediate filament organization, while cluster 2 was related to steroid and lipid metabolism and extracellular vesicle transport.

### Ultrastructure of mature oocytes is modified by MII-ffEVs treatment

**Figure 5.**
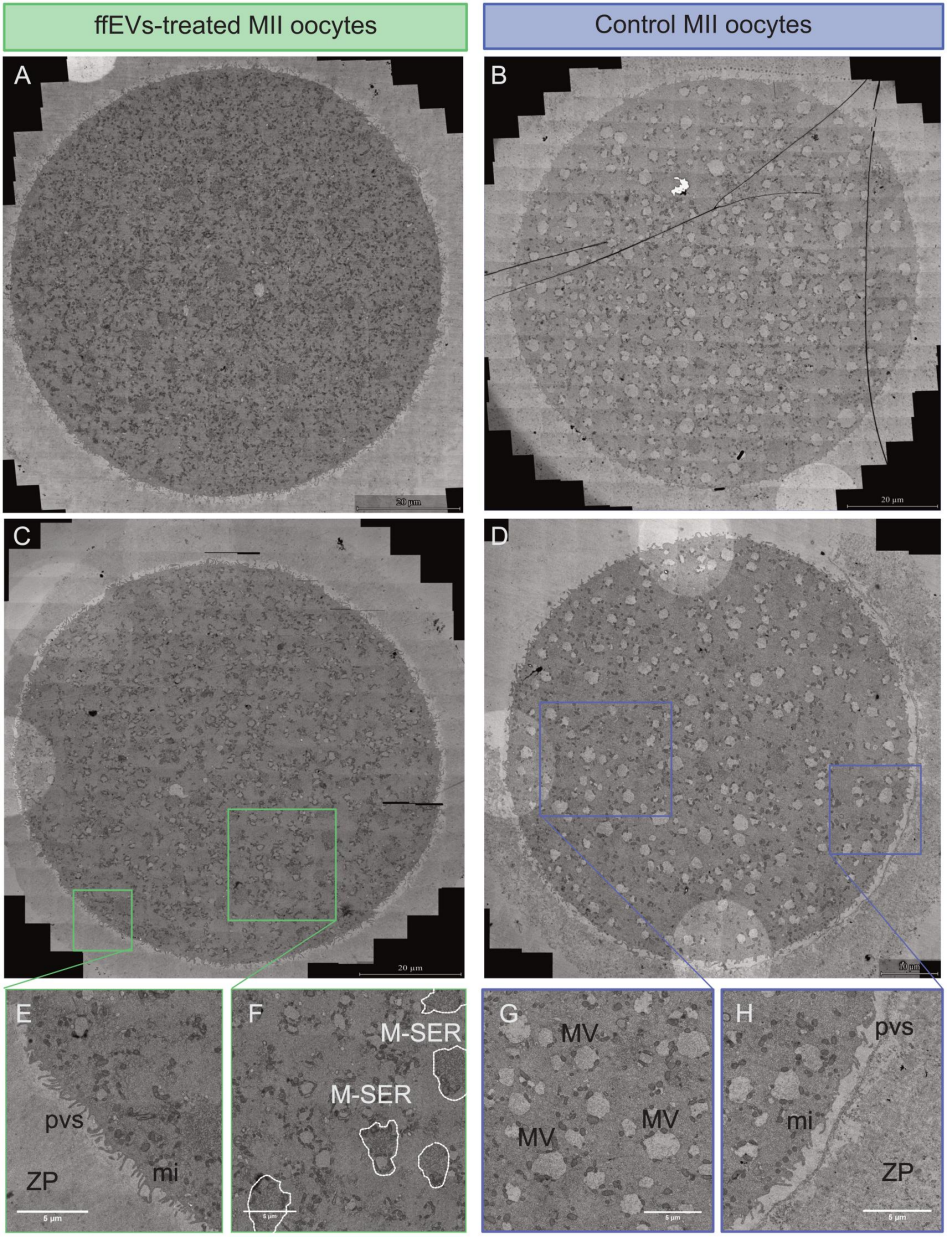
**Cytoplasmic reorganisation of oocytes treated or not with metaphase II-follicular fluid extracellular vesicles (MII-ffEVs) during *in vitro* maturation.** TEM images of oocytes. Scale bars (**A–C**) 20 µm, (**D**) 10 µm and (**E–H**) 5 µm. M-SER, mitochondria smooth endoplasmic reticulum (highlighted by white dotted line); MV, mitochondria vesicle; pvs, perivitelline space; mi, microvilli; ZP, zona pellucida.

TEM ultrastructural analysis of single oocytes revealed differences in oocyte organelle distribution and appearance between ffEVs-treated (Fig. 5A and C) and control mature oocytes (Fig. 5B and D). In particular, we observed differences in the appearance of smooth endoplasmic reticulum (SER) elements associated with mitochondria, called M-SER aggregates, and vesicles of SER and mitochondria, named MV complexes. While M-SER aggregates were more abundant in ffEVs-treated mature oocytes (Fig. 5F), the MV complexes at various sizes were more abundant in control mature oocytes (Fig. 5G). Treated oocytes had a more loose, microfilamentous microvilli architecture (Fig. 5E), while in control oocytes, microvilli appeared slightly shorter in length, fewer in number, and more irregularly distributed (Fig. 5H).

## Discussion

For the first time, we demonstrate here that EVs originating from human ovarian follicles can support oocyte maturation. First, we have identified the proteins present in ffEVs originating from follicles containing immature and mature oocytes, which provide promising targets for the development of supplements aimed at enhancing global IVM solutions. Second, our data suggest that these ffEVs proteins may be functionally significant, as evidenced by the internalisation of ffEVs by immature oocytes and a concomitant increase in oocyte maturation rates enhanced by 20%. Third, the interaction between the follicular milieu and oocytes during maturation, mediated by ffEVs, appears to induce substantial changes in the maturing oocytes. These changes are reflected in both the proteomic profile and ultrastructural alterations, with possible implications for oocyte competence. Below, we elaborate on these aspects and demonstrate how they collectively support the hypothesis that incorporating ffEVs can be integrated into any IVM protocol.

### The proteomic landscape of ffEVs derived from mature and immature follicles

All cells have the capability to secrete EVs. Studies in cattle have demonstrated that the proteomic cargo of ffEVs most likely originates from diverse cells in the ovarian microenvironment, including granulosa cells, theca cells, and cumulus cells, next to the oocyte itself (Uzbekova et al., 2020). In addition, ffEVs may originate from the blood plasma, as follicular fluid is formed in part via the transudation of plasma components. The role of ffEVs in the bidirectional oocyte–cumulus cell communication during follicular development is established as complementary to the traditional pathways mediated by transzonal projections (TZPs) and gap junctions (Machtinger *et al.*, 2016). Our comprehensive proteomic profiling of ffEVs originating from follicles containing mature and immature oocytes revealed an intriguing protein signature relevant to follicular homeostasis, encompassing essential protein machinery involved in steroid biosynthesis, endoplasmic reticulum organization, oxidoreductase activity and extracellular vesicle transport.

Despite the similar proteomic landscape between GV- and MII-ffEVs, we could highlight four proteins exclusively abundant in the MII-ffEVs cargo (F12, C1QC, IGKV1-39, and FREM-2). The roles of these proteins in the context of follicular biology suggest a link to oocyte competence (Anderson *et al*., 1993; Turathum *et al*., 2018; Reichhardt *et al*., 2019; Liu *et al.*, 2020; Poulsen *et al*., 2020; Zhou *et al*., 2021; Pla *et al*., 2022; Wang *et al*., 2022; Yu *et al*., 2023).

On the other side, proteins enriched in GV-ffEVs are associated with steroid biosynthesis and lipid metabolism, including CYP17A1 and CYP51A1. These proteins play a role in the activation of follicular fluid meiosis-activating sterol (FF-MAS), which promotes meiotic resumption when added to an IVM culture (Guo *et al.*, 2020). It is not unlikely that these proteins are introduced to the oocyte via ffEVs to facilitate GV oocyte maturation. Altogether, we pinpoint the proteins identified by our proteomic analysis in the GV- and MII-ffEVs for further investigation as mediators of follicle development, oocyte maturation, and competence. Importantly, we observed inter-patient variability in the abundance of DAPs within ffEVs, indicating that ffEVs from different patients may differ in their biological efficacy (Supplementary Fig. S2, Supplementary Table S12). While this variability could limit the effectiveness of autologous ffEV supplementation in some cases, it also provides an opportunity to refine future applications. Specifically, donors with potent ffEVs could be identified and included in a ‘superdonor’ bank for heterologous use, or, once the key cargo responsible for oocyte-supportive effects is characterized, synthetic ffEVs could be engineered to replicate and standardize this activity. Such strategies could mitigate biological variability and enhance the consistency and effectiveness of ffEV-based IVM protocols. Any ‘donor’ would undergo the same rigorous and routine screening tests already required for other donations, ensuring that the methodology will align with existing, high-standard safety procedures in reproductive medicine.

### Supplementation of rescue IVM culture with ffEVs increases oocyte maturation rate

The biological activity of EVs relies on the capacity of target cells to internalize them. It is proposed that EVs enter cells and release their cargo via endocytosis, phagocytosis, micropinocytosis, or direct fusion with the plasma membrane (Mulcahy *et al.*, 2014). EVs uptake studies in oocytes in different animal species have reported the presence of stained ffEVs in the cumulus cells (Hung *et al.*, 2015; de Almeida Monteiro Melo Ferraz *et al.*, 2020) and in the interface of oocytes with the cumulus cells, in the zona pellucida, and the ooplasm (da Silveira *et al.*, 2017; Lange-Consiglio *et al.*, 2017; Uzbekova *et al.*, 2020; Gabryś *et al.*, 2022). We demonstrated for the first time that cumulus cells denuded human oocytes can uptake labelled ffEVs, which can be detected in the zona pellucida after 24 h of co-incubation. Similar studies have been conducted in multiple animal models using COCs in the context of standard IVM. Two confocal studies in cat and cattle detected labelled ffEVs in the cumulus cells but not in the oocytes after 16–18 h of co-incubation with COCs (Hung *et al.*, 2015; de Almeida Monteiro Melo Ferraz *et al.*, 2020). However, in five other studies conducted using mice, canine, mare, and cattle COCs, positive ffEVs staining was detected in the interface of oocytes with the cumulus cells, in the zona pellucida, and the ooplasm after periods of incubation, raging from 15 to 72 h (da Silveira *et al.*, 2017; Lange-Consiglio *et al.*, 2017; Uzbekova *et al.*, 2020; Gabryś *et al.*, 2022; Fiorentino *et al.*, 2024). Our confocal microscopy findings locating ffEVs in the ZP are intriguing in this regard, as they raise the possibility of ffEVs being transferred into the oocyte via the cytoplasmic TZPs. TZPs are thin cytoplasmic extensions originating from cumulus cells that traverse the 3–5 µm thick zona pellucida to form gap-junctions and adherens-junctions with the oocyte, essential for communication between the oocyte and cumulus cells. Electron microscopy studies have detected EVs between the tip of the TZP and the oocyte plasma membrane (Macaulay *et al.*, 2014; Marchais *et al.*, 2022), suggesting a potential mechanism for transferring large cargo from granulosa cells to the oocyte. However, we have utilized denuded oocytes, which lack cumulus cells, and thus far, it is unknown whether TZPs’ tracks remain in the zona pellucida and could attract supplemented ffEVs. Our findings confirm that ffEVs likely transfer EVs cargo from the ovarian follicular fluid to the interior of the oocyte, but the precise mechanism mediating this internalization and the relevance of this transfer of molecular information remains unknown.

Performing rIVM in the presence of ffEVs resulted in an over 20% higher maturation rate, which is in line with a similar study in mares, whereby the supplementation of standard IVM culture with ffEVs resulted in a significant 25% increase in oocyte maturation rate (Gabryś *et al.*, 2022). A plethora of animal studies, but never human so far, have demonstrated the beneficial role of ffEV supplementation to standard IVM culture in terms of oocyte maturation and competence to produce good quality blastocysts (da Silveira *et al.*, 2017; Rodrigues *et al.*, 2019; de Almeida Monteiro Melo Ferraz *et al.*, 2020; Gabryś *et al.*, 2022; Han *et al.*, 2024). Currently, the mechanisms how ffEVs might contribute to improved oocyte maturation and competence in a standard IVM system is still not understood. However, it has been hypothesized that ffEVs might initially inhibit meiosis resumption in a similar way to the CNP peptide in the CAPA-IVM system (Pioltine *et al.*, 2020). The mechanism of ffEVs might be different from the one of the CNP peptide; the CNP-NPR2 modulates the cGMP levels, while the cargo of ffEVs might modulate the cAMP levels in the oocyte. More recently, a study suggested that ffEVs enhance IVM outcomes via the ERK1/2 pathway, which in turn results in an increase in the levels of ER and a decrease in ROS in porcine oocytes (Han *et al.*, 2024).

### The impact of the interaction of oocytes with ffEVs during oocyte maturation

For a deeper insight into how ffEVs contribute to improving oocyte maturation rates, we analysed oocytes subjected to rIVM with ffEVs using single-cell proteomics. Our methodology identified 4589 proteins present in the individual oocytes. The patients' intrinsic variability and the prolonged *in vitro* culture might have hidden some significant differences between ffEVs treated and control oocytes. Nevertheless, we identified differential abundant proteins between ffEVs-treated and control oocytes associated with keratinization, cytoskeleton organization, endopeptidase inhibitor activity, humoral immune responses, and, expectedly, extracellular vesicle transport. The organization of keratin and the cytoskeleton is critically important during oocyte maturation, with distinct differences in keratin distribution patterns observed between immature and mature oocytes (Fragouli *et al*., 2010; Kabashima *et al.*, 2010). Moreover, the intermediate filaments formed by keratins in oocytes play a significant role in early embryonic divisions and development through a mechanism in which differential keratin regulation impacts cell lineage fate (Lim *et al.*, 2020).

Amongst the proteins exhibiting higher expression in MII oocytes after ffEV-rIVM and also detected in ffEVs were skin-specific protein 32 (XP32), filaggrin-2 (FLG2), and protein-glutamine gamma-glutamyltransferase E (TGM3). The literature identifies these three proteins as key maternal proteins participating in healthy embryo development (Iqbal *et al.*, 2014; Foresta *et al.*, 2016; Virant-Klun *et al.*, 2016; Zha *et al.*, 2023).

It is well established that EVs have the capacity to indirectly decrease the synthesis of proteins by decreasing their corresponding transcripts through their EV-miRNA cargo. It has been reported that the expression of most of the proteins with lower abundance in our dataset is regulated by miRNAs, of which miR-223-3p, miR-148a-3p, hsa-let-7a-5p, and hsa-let-7b-5p have been found in the ffEVs in our study. Other studies have shown that injecting specific miRNAs in oocytes can exert changes in mRNA translation and, hence, impact protein availability (Kataruka *et al.*, 2020). Notably, the protein with the largest drop in abundance after supplementation with ffEVs, BRI3-binding protein (BRI3BP), which is localized to the mitochondria and the ER, is downregulated to avoid developmental arrest at the morula stage (Nõmm *et al.*, 2023).

Of particular interest is the presence of hyaluronan synthase 1 (HAS1), the most differentially abundant protein in MII oocytes undergoing ffEV-facilitated rIVM, intriguingly absent in the ffEVs themselves indicating no direct transfer from ffEVs. There is a substantial amount of literature on the role of hyaluronan synthesis during oocyte maturation and competence establishment (Fülöp *et al.*, 1997; Kimura *et al.*, 2002; Stock *et al.*, 2002; Schoenfelder and Einspanier, 2003; McKenzie *et al.*, 2004; Siiskonen *et al.*, 2015). Yokoo and Sato reported that hyaluronan synthesis and accumulation may be involved in the expansion of COC’s volume and play an important role in the progression of meiotic resumption and the induction of oocyte maturation (Yokoo and Sato, 2011). In addition, HAS1 has been associated with the endoplasmic reticulum (Siiskonen *et al.*, 2015), explaining a hypothetical connection between the increased abundance of HAS1 and increased endoplasmic reticulum structures in ffEV-treated oocytes, which calls for further investigations.

To translate our findings into hypotheses elucidating the mechanisms of ffEV-facilitated rIVM, we explored the proteomic landscape of the ffEV–oocyte communication network, identifying potential candidates that merit further investigation. Hence, we observed two intriguing clusters, of which one is solely relevant to oocyte activity, and another integrates the actions of both oocytes and ffEVs. It is tempting to hypothesize that ffEVs deliver cargo involved in steroid and lipid metabolism to GV oocytes, facilitating meiotic resumption via modulation of FF-MAS, which promotes meiotic resumption when added to IVM medium (Guo *et al.*, 2020). Concurrently, oocytes may fine-tune their keratin distribution and intermediate filament organization to prepare for maturation in an environment conducive to fertilisation. We highlight specific proteins that warrant exploration in mechanistic studies, particularly CYP17A1, HSD3B2, CYP51A1, MSMO1, SCD, SLC27A1, ESYT1, and APOB as involved proteins in interactions between the oocyte and ffEVs in steroid synthesis and lipid metabolism.

### Ultrastructure of oocytes after ffEV-rIVM

It was intriguing to observe such a striking difference in the ratio of MV/M-SER between the two groups of mature oocytes. M-SER is an ultrastructure exclusively observed in mature oocytes, and a moderate number of M-SER with MVs in the ooplasm is a common feature of healthy mature oocytes (Palmerini *et al.*, 2014). The higher abundance of MVs—larger or dilated SER vesicles in the ooplasm—in oocytes not subjected to ffEV supplementation could be a sign of ER stress (Miglietta *et al.*, 2023). Indeed, dilatation and vacuolization of ER cisterns were a result of the treatment of oocytes with an inductor of ER stress (Vašíčková *et al.*, 2018). In contrast, the presence of numerous and large ooplasmic MV complexes in human mature oocytes, caused by swelling and coalescence of isolated SER vesicles, were stipulated to indicate cytoskeletal defects (Gualtieri *et al.*, 2009). Dilatation and vesiculation of ER, associated with the uncoupling/loss of associated mitochondria, have also been observed in human-aged oocytes and correlated to oxidative stress (Oslowski and Urano, 2011; Bianchi *et al.*, 2015). Similar to our study, Bianchi et al. additionally observed fewer M-SER aggregates and increased swelling of SER tubules in aged oocytes, but also associated with long *in vitro* culture (48 h) or the components of the *in vitro* milieu (Bianchi *et al.*, 2015). The TEM observations regarding altered SER structures are in line with oocyte proteomic data pointing to several DAPs related to ER stress (BRI3BP, CANX, HSP90B1, PDIA4, PDIA6, PDIA3, HSPA5, ERLIN2, ERP44, UGGT1, ERMP1, TMX1). Overrepresentation of MVs in MII oocytes from rIVM without ffEVs could compromise the probability of fertilisation since SER plays a key role in calcium storage and release at oocyte activation during fertilisation (Whitaker, 2006). Moreover, changes in complexes of SER and associated mitochondria could induce a major impact on the oocyte in terms of energy accumulation, protein and lipid production, and production of nuclear membranes throughout early embryo development (Carroll, 1996). Besides the MV-M-SER ratio, we observed differences in the zona pellucida and microvilli morphology between the two groups of mature oocytes. While ffEV-supplemented oocytes had a loose, microfilamentous architecture, controls showed a more dense and granular texture. Regarding the microvilli pattern, literature findings imply that mature oocytes with normal morphology have numerous long and thin microvilli extending into the perivitelline space, an observation in line with the microvilli we noted in ffEVs-supplemented oocytes (Palmerini *et al.*, 2014). These differences in microvilli morphology might affect the fertilization *a posteriori* since microvilli are suggested to play a role during fusion between the oocyte and the sperm (Runge *et al.*, 2007). A deep understanding of these ultrastructural changes will inform and ensure the biosafety and clinical utility of oocytes following r-IVM with ffEVs.

Our study is subject to certain limitations that may have introduced interpretational biases. First, the oocytes were initially vitrified at the GV stage and subsequently warmed for experimentation, implying that potential cryodamage cannot be ruled out for both treated and untreated oocytes. Some ultrastructural observations may be attributable to the vitrification and warming procedures, as noted by Palmerini *et al.* (2014). Additionally, we performed uptake assays using a system that employs a green fluorescent dye to indicate ffEV uptake by oocytes. However, the ooplasm and certain oocyte features, such as refractile bodies, tend to display autofluorescence, introducing an unspecific signal that is nevertheless distinguishable from the specific signal of ffEV uptake (Otsuki *et al.*, 2007). Although proteomics and TEM studies provided insight into the potential competence of mature oocytes from rIVM, the ultimate test of competence—assessing fertilisation rate through insemination with ICSI—was not feasible due to legal restrictions. Finally, we invite other researchers to test our proposed ffEV-facilitated IVM system in alternative models, such as CAPA-IVM and standard IVM using unstimulated COCs, rather than denuded GVs from stimulated cycles, which may have inherent developmental limitations.

In conclusion, we propose a novel approach adaptable to global IVM systems, where ffEVs should be further investigated as an adjunct to enhance oocyte maturation rates and potentially improve oocyte competence. This possible competence enhancement requires further validation through parthenogenic activation or insemination of oocytes from IVM. Our findings hold the promise of directly benefiting patients seeking reproductive medicine. Once optimized for clinical application, IVM could offer significant advantages to subfertile women at risk of OHSS, cancer patients undergoing fertility preservation, and the broader population undergoing IVF. This advancement could facilitate a more streamlined, cost-efficient, and safer approach to infertility treatment, ultimately contributing to heightening of women’s health.

## Supplementary Material

hoag021_Supplementary_Data

## Data Availability

The mass spectrometry proteomics data have been now deposited to the ProteomeXchange Consortium via the PRIDE partner repository with the dataset identifier PXD073018.
