# Supplementary material for: Treatment of human oocytes with extracellular vesicles from follicular fluid during rescue in vitro maturation enhances maturation rates and modulates oocyte proteome and ultrastructure
Source: Hum Reprod Open. 2026 Mar 9;2026(2):hoag021. doi: 10.1093/hropen/hoag021 (PMC13037814; doi:10.1093/hropen/hoag021)
Supplement: hoag021_Supplementary_Data [file hoag021_supplementary_data.zip › Supplementary_Data__Revised_1002026_EO.docx]

**Supplementary Data**

**Supplementary Figures**


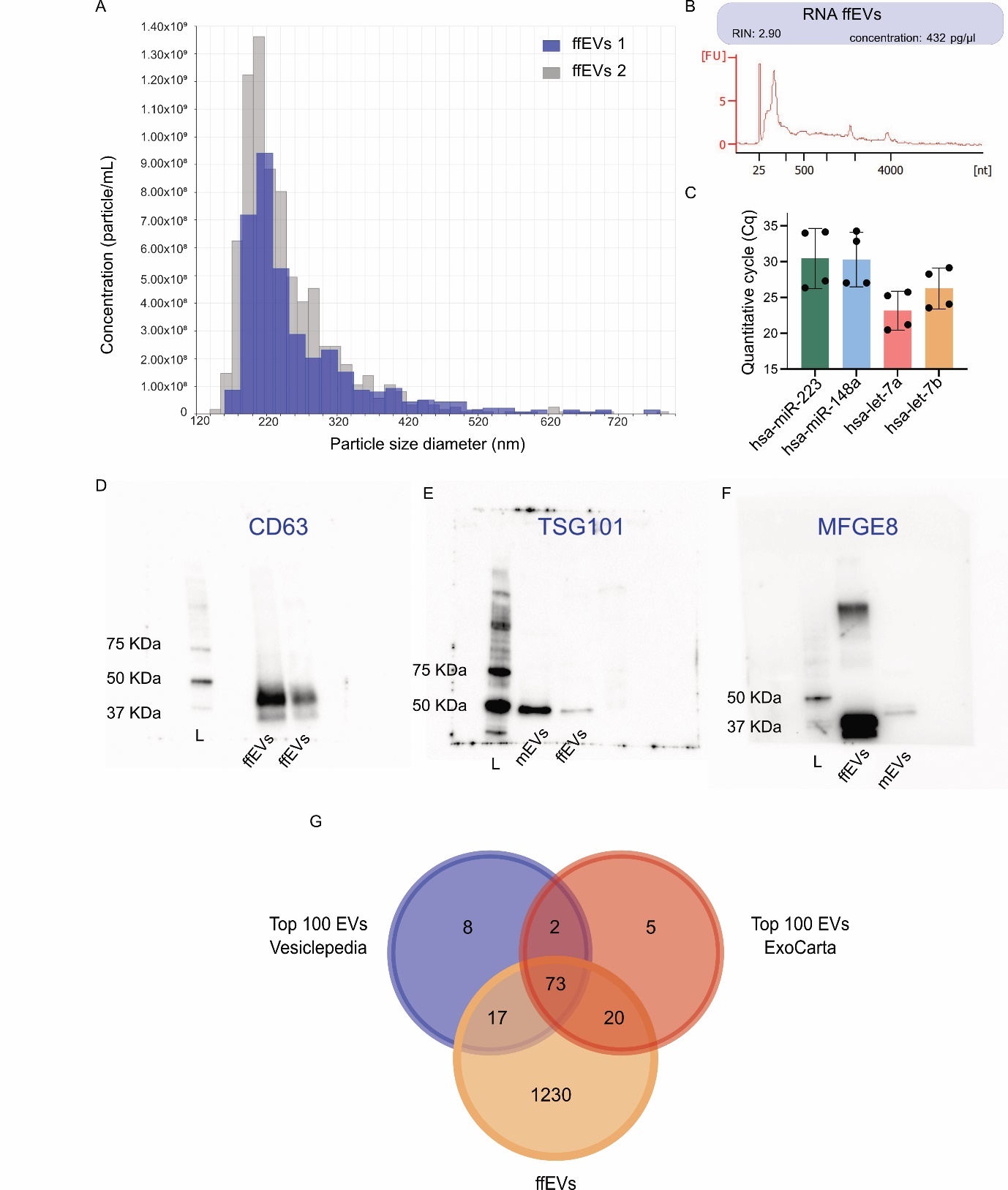


**Supplementary Figure S1 Size distribution evaluation** **unveiled particle dimensions ranging from 50 to 700 nm** (**A**). We successfully extracted measurable quantities of RNA from ffEVs derived from 1.5 – 2.0 mL of patient-individual follicular fluid. The Bioanalyzer profiles demonstrated that the majority of the RNA belonged to the small RNA population, with a minimal contribution from larger RNAs (**B**). Furthermore, within the isolated ffEVs, we identified the presence of typical follicular fluid miRNAs (**C**). Western blot analysis of the ffEVs samples confirmed their identity by detecting specific protein markers associated with EVs (**D–F**). Specifically, the transmembrane proteins CD63 and the cytosolic protein Tumor susceptibility gene 101 (TSG101) commonly enriched in EVs were present in our samples. L, Ladder; mEV, milk EVs.

**
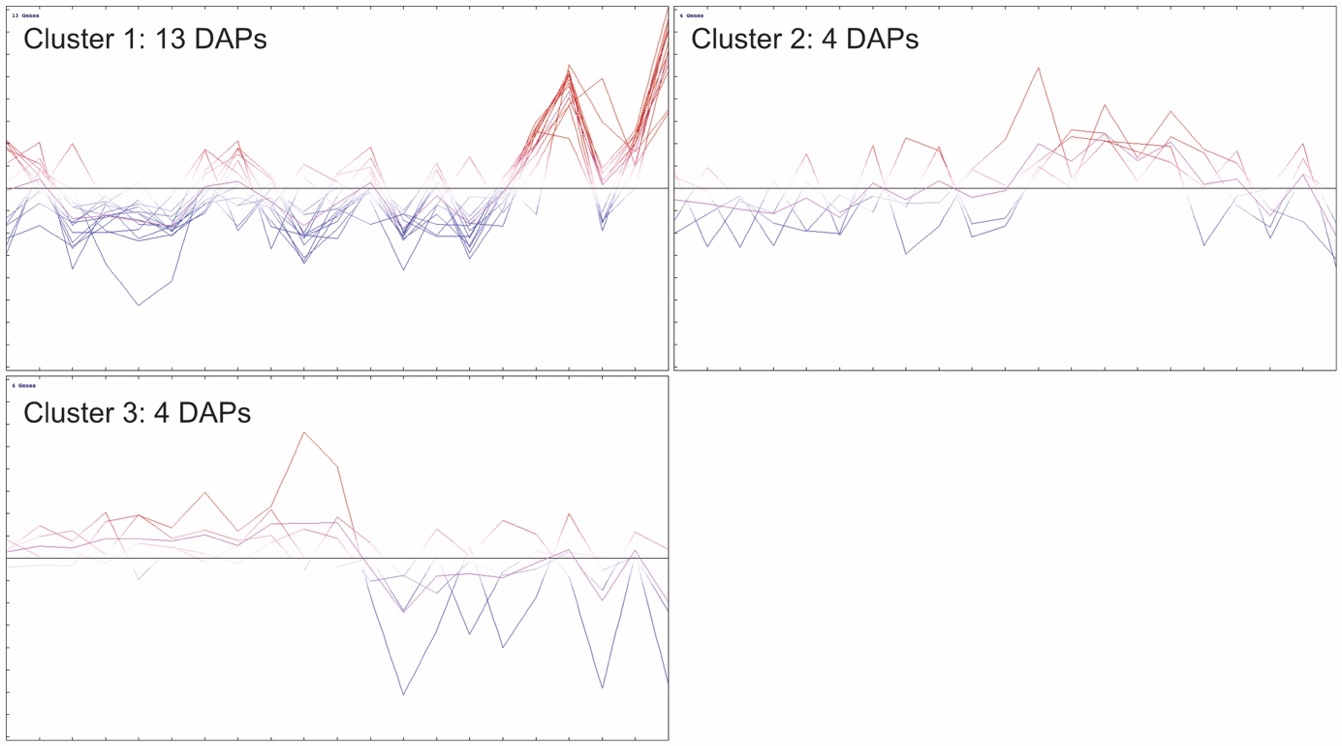
**

**Supplementary Figure S2 Expression graph for each one of the three clusters of DAPs arising from the SOTA analysis of ffEVs.** A principal component analysis (PCA) based on the top 50 proteins with the greatest changes across the dataset showed a dynamic protein ffEV pattern, with GV-ffEVs samples separated from the rest of the samples based on principal component 1. To reveal differential abundant proteins (DAPs), three comparisons were statistically performed: MII versus (vs.) GV, GVBD vs. MII, and GVBD vs. GV, resulting in 14, 7, and 1 DAPs, respectively. The DAPs resulting from the three comparisons were further analysed for their expression profiles across samples using a Self-Organizing Tree Algorithm (SOTA) clustering analysis. This analysis provided three clear clusters: cluster 1, with 13 proteins more abundant in GV-ffEVs; cluster 2, with 4 proteins more abundant in GVBD-ffEVs; and cluster 3, with 4 proteins more abundant in MII-ffEVs. Each individual line in red or blue represents one protein up or downregulated, and the pink line in the middle shows the average expression of all the proteins in each sample. The protein intensities for each cluster of DAPs have been provided in Supplementary Table S12.
